# Vegetative Reproduction Is More Advantageous Than Sexual Reproduction in a Canopy-Forming Clonal Macroalga under Ocean Warming Accompanied by Oligotrophication and Intensive Herbivory

**DOI:** 10.3390/plants10081522

**Published:** 2021-07-26

**Authors:** Hikaru Endo, Toru Sugie, Yukiko Yonemori, Yuki Nishikido, Hikari Moriyama, Ryusei Ito, Suguru Okunishi

**Affiliations:** 1 Faculty of Fisheries, Kagoshima University, Kagoshima 890-0056, Japan; toru90118@gmail.com (T.S.); k5454895@kadai.jp (Y.Y.); k5945870@kadai.jp (Y.N.); k0531012@kadai.jp (H.M.); okunishi@fish.kagoshima-u.ac.jp (S.O.); 2 United Graduate School of Agricultural Sciences, Kagoshima University, Kagoshima 890-0065, Japan; 3 Fisheries Research Division, Oita Prefectural Agriculture, Forestry and Fisheries Research Center, Bungotakada 879-0608, Japan; ito-ryusei@pref.oita.lg.jp

**Keywords:** climate change, foundation species, fucoid brown algae, non-additive effect, simulated herbivory

## Abstract

Ocean warming and the associated changes in fish herbivory have caused polarward distributional shifts in the majority of canopy-forming macroalgae that are dominant in temperate Japan, but have little effect on the alga *Sargassum fusiforme*. The regeneration ability of new shoots from holdfasts in this species may be advantageous in highly grazed environments. However, little is known about the factors regulating this in *Sargassum* species. Moreover, holdfast tolerance to high-temperature and nutrient-poor conditions during summer has rarely been evaluated. In the present study, *S. fusiforme* holdfast responses to the combined effects of temperature and nutrient availability were compared to those of sexually reproduced propagules. The combined effects of holdfast fragmentation and irradiance on regeneration were also evaluated. Propagule growth rate values changed from positive to negative under the combination of elevated temperature (20 °C–30 °C) and reduced nutrient availability, whereas holdfasts exhibited a positive growth rate even at 32 °C in nutrient-poor conditions. The regeneration rate increased with holdfast fragmentation (1 mm segments), but was unaffected by decreased irradiance. These results suggest that *S. fusiforme* holdfasts have a higher tolerance to high-temperature and nutrient-poor conditions during summer than propagules, and regenerate new shoots even if 1-mm segments remain in shaded refuges for fish herbivory avoidance.

## 1. Introduction

Plant reproduction can be divided into sexual and asexual reproduction; the latter includes somatic embryogenesis and vegetative reproduction, which occurs without embryo formation [1]. Vegetative reproduction is common in clonal plants, which produce new shoots (i.e., ramets) from roots, stolons, or rhizomes [2,3,4]. The newly produced ramets obtain resources, such as nutrients and carbohydrates, from physiologically integrated mother plants through connections [4]. Moreover, fragmentation of these ramets via the disturbance or senescence of these connections often enhances new ramet production [2,3]. Therefore, vegetative reproduction may be more advantageous than sexual reproduction under resource-limited and highly disturbed environments.

Brown, red, and green macroalgae are the major primary producers in coastal marine ecosystems. Specifically, the canopy-forming large brown algae, kelp (Laminariales) and fucoid species (Fucales), are highly productive and act as foundation species [5], providing food, habitats, and spawning grounds for various marine organisms [6,7]. Furthermore, the conservation and restoration of marine macroalgal forests, which export carbon to the deep sea, may contribute to the mitigation of climate change caused by an increase in the atmospheric CO_2_ level [8,9]. However, these algal forests have been declining due to ocean warming [10]. Above-average temperatures, combined with nutrient-poor conditions during the summer, have been known to cause physiological stress in macroalgal species [11,12,13,14].

Moreover, ocean warming has caused a range expansion of tropical herbivorous fishes into temperate waters, resulting in an increase in their grazing activity, especially in ocean warming hotspots, such as the Mediterranean and southern Japan [15]. Additionally, in the western North Pacific around southern Japan, nutrient concentrations in the surface mixed layer have been declining because the mixing of nutrient-poor surface water and nutrient-rich deep water has been suppressed by ocean warming or longer-term natural climate change [16]. Consequently, the majority of the kelp and fucoid species that are dominant in temperate Japan have shifted their distributional range towards the pole [17]. However, such poleward range shifts have not been observed in the fucoid *Sargassum fusiforme* [17], implying that this species might have reproductive traits that allow for its survival in warm, nutrient-poor, and highly grazed environments in southern Japan.

*Sargassum* species generally have perennial holdfasts (analogous to rhizoids), stipes (analogous to stems), and annual shoots (i.e., main branches), which show large seasonal variations in biomass and length, with the exception of annual species such as *S. horneri* [18,19,20,21]. In temperate *Sargassum* species, including *S. fusiforme*, these shoots commonly germinate from stipes during summer, grow between autumn and spring, and decay during the subsequent summer after the production of propagules via sexual reproduction [18,20]. Moreover, vegetative reproduction via the regeneration of new shoots from holdfasts has been reported in several *Sargassum* species, including *S. fusiforme* [22,23,24]. In southern Japan, where fish herbivory is intensive between summer and autumn, and is weaker during winter [25], *Sargassum* shoots derived from holdfasts or propagules only grow from winter to spring, and decay during summer [26,27,28]. Therefore, these propagules and holdfasts, rather than the shoots, are exposed to warm and nutrient-poor conditions during summer. Previous studies have shown the effect of increased temperature on propagule growth [29,30], and the combined effects of temperature and nutrient availability or salinity on shoot growth [31,32,33] in *Sargassum* species. However, the combined effects of elevated temperature and reduced nutrient availability on the growth of propagules and holdfasts have rarely been evaluated; therefore, it is unclear whether sexual or vegetative reproduction is more advantageous under warm and nutrient-poor environments.

Moreover, Ito et al. [22] showed that the regeneration of new shoots from holdfasts was enhanced by cutting the filamentous holdfasts into segments <2.5 mm in length in *S. fusiforme*. This implies that this species might regenerate even after the holdfasts are fragmented by fish herbivory, as reported in *S. swartzii* [24]. They also reported that the percentage of *S. fusiforme* holdfasts that regenerated new shoots tended to increase in response to elevations in temperature (from 17 °C to 23 °C) and irradiance (from 50 to 230 µmol photons m^−2^ s^−1^) [22]. However, the effects of a broader range of temperatures and nutrient availability on regeneration have not been studied and therefore the most important factor regulating regeneration is unclear.

Furthermore, microtopographic refuges, such as crevices, are known to enhance the recruitment and survival of *Sargassum* propagules in tropical regions, where intensive fish herbivory occurs [34]. Although these microhabitats are predicted to act as holdfast refuges from fish herbivory, reduced light availability in a shaded crevice may antagonize the positive effect of holdfast fragmentation by fish herbivory. However, the combined effects of fragmentation and decreased irradiance on regeneration are unclear based on the results of single-factor studies.

*Sargassum fusiforme* is common between lower intertidal and upper subtidal reefs in Japan, China, and Korea [35,36]. This species is edible and has been cultivated in these countries. Due to its commercial importance, several studies on the ecological and physiological traits of this species have been conducted [20,22,33,37], although the effects of ocean warming, nutrients, and herbivory on its reproductive traits have not been examined. Novel knowledge of the reproductive traits of this species may improve seeding methods for its cultivation.

In the present study, four laboratory culture experiments of *S. fusiforme* were conducted to examine (1) the combined effects of temperature (10 °C–30 °C) and nutrient availability on propagule growth, (2) the combined effects of temperature (15 °C–30 °C) and nutrient availability on the growth and regeneration rates of holdfasts, (3) the effect of high temperature (30 °C–38 °C) on the growth and regeneration rates of holdfasts, and (4) the combined effects of holdfast fragmentation and irradiance on the growth and regeneration rates of holdfasts in this species.

## 2. Results

### 2.1. Experiment 1: Combined Effects of Temperature and Nutrients on Propagules

**Figure 1:**
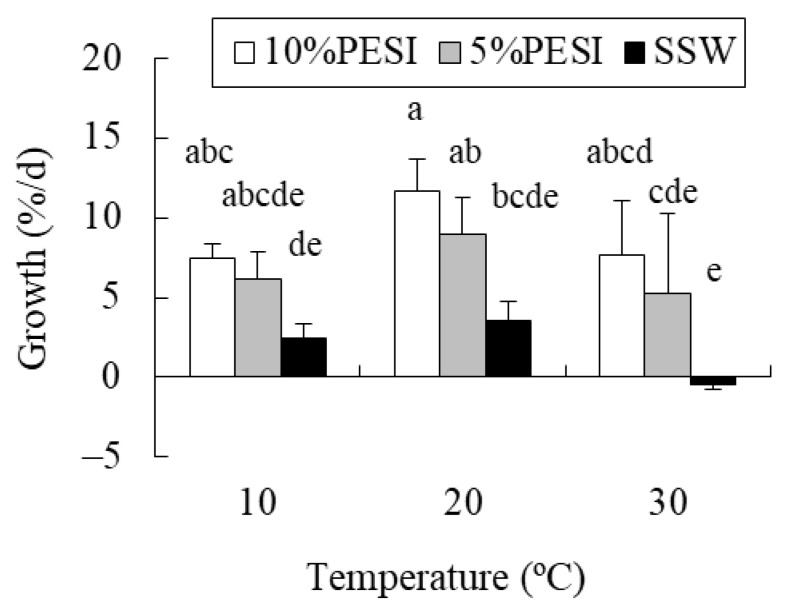
Relative growth rate of *Sargassum fusiforme* propagules cultured in nine different treatments (Mean + SD, *n* = 6). Different small letters indicate statistical significance among different treatments (*p* < 0.05).

Mean (± standard deviation) dissolved inorganic nitrogen (DIN) concentrations in 10% Provasoli’s enriched seawater (PESI), 5% PESI, and sterile natural seawater (SSW) were 106.36 ± 1.07 µM, 74.89 ± 7.63 µM, and 6.94 ± 0.17 µM, respectively. The two-way analysis of variance (ANOVA) detected significant effects of temperature and nutrients, and their interaction on the relative growth rate of propagules (Table 1). The results of Tukey’s test showed that there was no significant difference in the growth rate among temperatures in 10% PESI treatments, whereas the values decreased in response to elevated temperature from 20 °C to 30 °C in the 5% PESI treatment (Figure 1). The growth rate was significantly lower in non-enriched SSW treatments than in 10% PESI treatments at all temperatures. Moreover, the positive growth rate became negative in response to temperature elevation from 20 °C to 30 °C in the SSW treatment.

### 2.2. Experiment 2: Combined Effects of Temperature and Nutrients on Holdfasts

**Figure 2:**
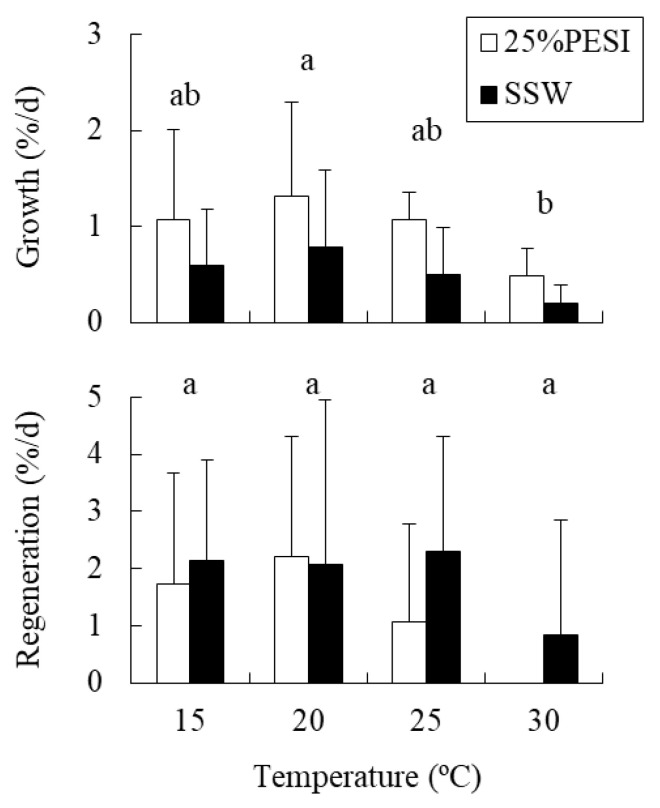
Relative growth and regeneration rates of *Sargassum fusiforme* holdfasts cultured in eight different treatments (Mean + SD, *n* = 6). Different small letters indicate statistical significance among different temperature treatments (*p* < 0.05).

The mean (± standard deviation) DIN concentrations in 25% PESI and SSW were 150.47 ± 1.18 µM and 5.26 ± 2.70 µM, respectively. The holdfast relative growth rate was significantly affected by temperature and nutrient availability, although their interaction was not significant (Table 2). The growth rate was significantly lower at 30 °C than at 20 °C, and lower in SSW treatments than in 25% PESI treatments (Figure 2). In contrast, the relative regeneration rate was unaffected by both temperature and nutrients, although values tended to decrease in response to elevated temperature from 20 °C to 30 °C, especially in the 25% PESI treatment.

### 2.3. Experiment 3: Effect of High Temperature on Holdfasts

**Figure 3:**
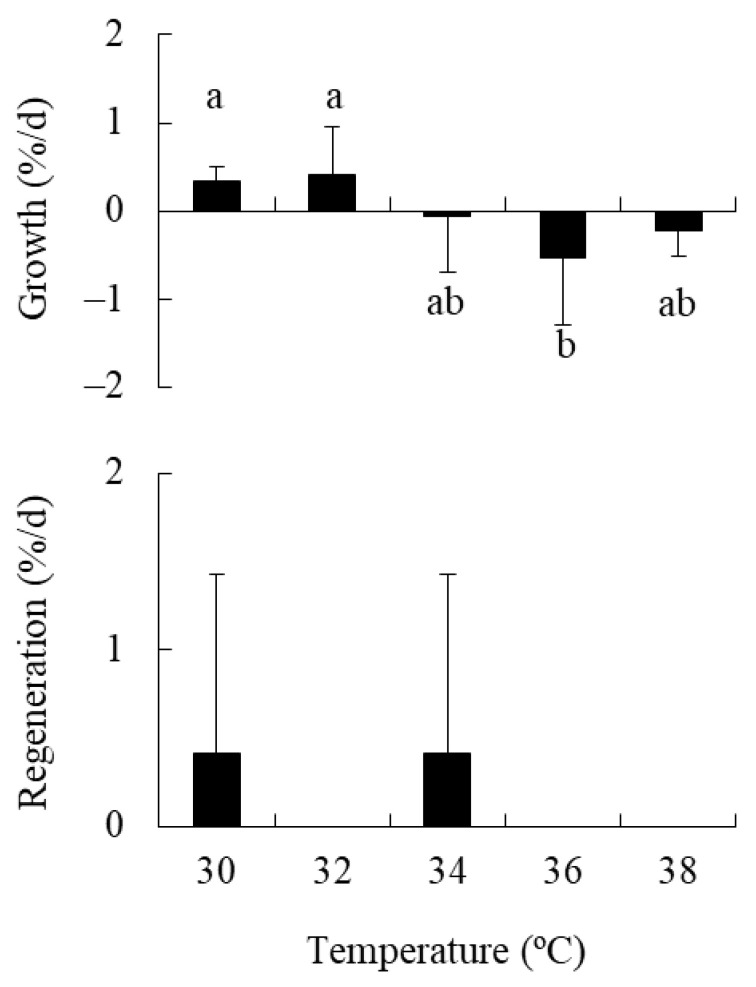
Relative growth and regeneration rates of *Sargassum fusiforme* holdfasts cultured in five different temperatures (Mean + SD, *n* = 6) using SSW as the culture media. Different small letters indicate statistical significance among different temperature treatments (*p* < 0.1).

A significant effect of temperature (between 30 °C and 38 °C) on the relative growth rate of holdfasts was detected by one-way ANOVA (df = 4, MS = 0.859, *F* = 2.998, *p* = 0.040). Tukey’s test indicated that the growth rate was higher at 30 °C and 32 °C than at 36 °C under a significance level of *p* < 0.1 (Figure 3), although the differences among temperatures were not detected under a *p* < 0.05 level. The mean value was positive at 30 °C–32 °C and was negative at 34 °C–38 °C. In contrast, the relative regeneration rate was not significantly affected by temperature (df = 4, MS = 0.306, *F* = 0.75, *p* = 0.567). Regeneration was even observed at 30 °C and 34 °C, but not in any other treatments.

### 2.4. Experiment 4: Combined Effects of Fragmentation and Irradiance on Holdfasts

**Figure 4:**
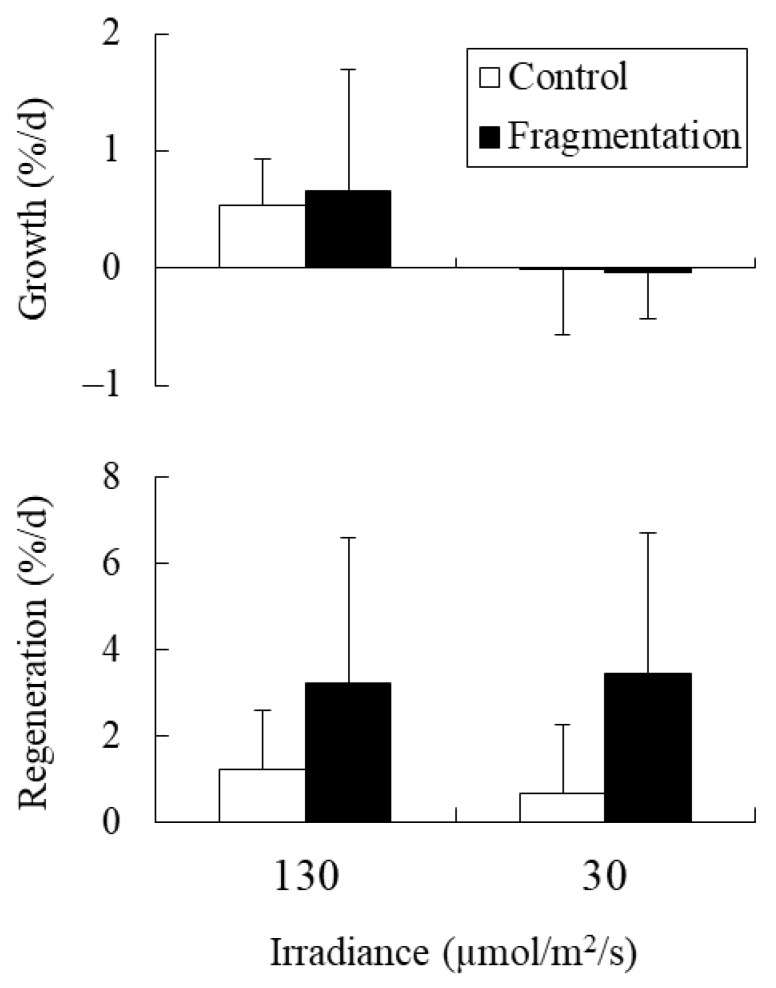
Relative growth and regeneration rates of *Sargassum fusiforme* holdfasts cultured in eight different treatments (Mean + SD, *n* = 6) at 30 °C using SSW as the culture media.

The holdfast relative growth rate was significantly affected by irradiance, but not by fragmentation or their interaction (Table 3). Values decreased in response to reduced irradiance from 130 to 30 µmol photons m^−2^ s^−1^ (Figure 4). In contrast, the relative regeneration rate was significantly affected by fragmentation, but not by irradiance or their interaction (Table 3). Fragmentation significantly increased the regeneration rate, even in the low-irradiance treatments (Figure 4).

## 3. Discussion

Baba [29] examined the combined effects of seven temperature levels (10 °C, 15 °C, 20 °C, 25 °C, 30 °C, 32 °C, and 34 °C) and four irradiance levels (10, 25, 100, and 180 µmol photons m^−2^ s^−1^) on the relative growth rate of *S. fusiforme* propagules in a 20-d experiment using 100% PESI (DIN = ca. 800 µM) as a culture medium. Growth rates were reported to be the highest at 25 °C–30 °C at 100–180 µmol photons m^−2^ s^−1^. The present 21-d study evaluated the combined effects of three temperature levels (10 °C, 20 °C, and 30 °C) and three nutrient levels (10% PESI, 5% PESI, and SSW) on the growth rate of *S. fusiforme* propagules, and found a significant interaction between temperature and nutrient availability. Negative effects of increased temperature from 20 °C to 30 °C were not detected in 10% PESI treatments (DIN = ca. 100 µM), but were found in 5% PESI treatments (DIN = ca. 75 µM). Moreover, the growth rate in non-enriched SSW treatments (DIN = ca. 7 µM) was lower than those in 10% PESI treatments at all temperatures, and changed from positive to negative values in response to a temperature elevation from 20 °C to 30 °C. These results suggest that the high-temperature tolerance of *S. fusiforme* propagules strongly depends on nutrient availability. Similar results have been obtained in our previous studies using juvenile sporophytes of the kelps *Undaria pinnatifida*, *Ecklonia cava*, and *Sacchrina japonica* [12,13,14]. Therefore, the early life stages of kelp and fucoid species appear to be vulnerable to reduced nutrient availability, especially under warm conditions, probably because of their small size and limited resource accumulation.

Yatsuya et al. [23] reported that holdfasts of *Sargassum piluriferum* and *S. alternato-pinnatum* regenerated new shoots after incubation at 32.5 °C for 5–17 d. In the present study, there were significant effects of temperature and nutrient availability on the growth of *S. fusiforme* holdfasts, although there was no significant interaction between the two factors, indicating an additive effect. Holdfast growth decreased in response to elevated temperature from 20 °C to 30 °C and reduced nutrient availability from 25% PESI (DIN = ca.150 µM) to SSW (DIN = ca.5 µM). However, the growth rate remained at positive values even at 30 °C in nutrient-poor SSW treatment, in contrast to the results of the propagules. Moreover, the growth rates maintained positive values at 32 °C, and became negative at 34 °C in our subsequent 28-d experiment using SSW as a culture medium, whereas the propagules decreased their growth rate in response to the temperature elevation from 30 °C to 32 °C and withered within 4 d at 34 °C under nutrient-rich 100% PESI conditions [29]. These results suggest that the holdfasts of this species have a higher tolerance to high-temperature and nutrient-poor conditions during summer than propagules of the same species. High-temperature tolerance of marine macroalgae is known to be associated with the ability to accumulate and maintain an internal nitrogen reserve [11,38,39]. Hence, the holdfasts, which are larger than the propagules, may withstand warm and nutrient-poor conditions using stored nitrogen.

Ito et al. [22] showed that the number of regenerated shoots per holdfast length was higher for holdfasts that were fragmented into lengths of 1 mm than for the longer holdfasts (i.e., 2.5, 5, 10, and 20 mm in length) in *S. fusiforme*. Similarly, in the present study, the regeneration rate significantly increased with holdfast fragmentation from 5 mm to 1 mm in length. Ito et al. [22] also reported that the percentage of *S. fusiforme* holdfasts that geminated new shoots tended to decline in response to decreased temperature (from 23 °C to 17 °C) and irradiance (from 230 to 50 µmol photons m^−2^ s^−1^). However, in the present study, the regeneration rates of new shoots from *S. fusiforme* holdfasts were not significantly affected by broader ranges of temperature (15 °C–30 °C and 30 °C–38 °C), nutrient availability, or irradiance, although the values tended to decrease in response to temperature elevation from 20 °C and 30 °C and nutrient enrichment. These results indicated that the regeneration of new shoots from holdfasts of this species is strongly regulated by physical stimulation (i.e., fragmentation) rather than abiotic environmental factors. This enhancement of vegetative reproduction by fragmentation is common in clonal plants and may be advantageous for reproduction in highly disturbed environments [2,3].

Loffler et al. [24] showed that the survival rate of *S. swartzii* was unaffected by the experimental removal (to mimic fish herbivory) of 50% of holdfast biomass but decreased when 75% of holdfast biomass was removed. In contrast, Ito et al. [22] and the present study showed that the filamentous holdfasts of *S. fusiforme* regenerated new shoots even after they were fragmented into 1-mm segments. Moreover, the present study showed that the regeneration rate was unaffected by reduced irradiance from 130 to 30 µmol photons m^−2^ s^−1^, and the positive effect of fragmentation was not antagonized by the irradiance reduction. Hence, the holdfasts of this species can regenerate new shoots if the tiny segments remain in shaded refuges, such as crevices [34], to avoid intensive fish herbivory. However, holdfast growth decreased in response to reduced irradiance in the present study. Therefore, the survival rate of the holdfasts may depend on the light environment of these refuges.

*Sargassum fusiforme* is distributed from Hokkaido in northern Japan to Okinawa Prefecture in southern Japan [35]. The maximum seawater temperature and DIN concentration ranges during summer (between July and September) are 28.2 °C–29.1 °C and 1.1–4.5 µM, respectively, at several sites in Kagoshima Prefecture [26,27], near the southern distributional limit of this species. The results of the present study predict that *S. fusiforme* holdfasts can grow during the summer in Kagoshima Prefecture, and have the potential to survive under a further warming of 2 °C–3 °C [40], whereas their propagule survival during summer depends on the local nutrient environment in this region. The present study also showed that holdfast fragmentation enhanced vegetative reproduction. These traits may allow survival under the warm, nutrient-poor, and highly grazed environments in southern Japan, where local extinctions of other kelp and fucoid species have been reported [17]. However, further genetic approaches are required to quantify the contribution of vegetative and sexual reproduction to population persistence, and to determine the genotype that enables the growth of sexually-reproduced propagules into matured individuals with large holdfasts that exhibit vegetative reproduction.

*Sargassum fusiforme* is a popular food in China, Korea, and Japan, and therefore seeding methods for the cultivation of this species have been developed [22,41,42,43]. Ito et al. [22] suggested that holdfasts harvested during spring can be stored by incubation at an irradiance of 1 µmol photons m^−2^ s^−1^ in order to suppress regeneration, and can be utilized for the seeds by means of a tank culture for 40 d after cutting them into segments <2.5 mm in length. This method seems to be more efficient and effective than the one based on sexual reproduction in southern Japan under warming, according to the results of the present study. However, little is known about differences in growth characteristics between regenerated shoots and sexually reproduced shoots. Further studies on the ecological and physiological traits of holdfasts and regenerated shoots of this species may provide insights for the conservation and restoration of marine macroalgal forests in southern Japan under ocean warming conditions, and for the improvement of seed production methods for this species.

## 4. Materials and Methods

### 4.1. Experiment 1: Combined Effects of Temperature and Nutrients on Propagules

*Sargassum fusiforme* propagules were cultured for 21 d in nine different treatments, consisting of three temperature levels (10 °C, 20 °C, and 30 °C) and three nutrient levels. These temperature levels were chosen based on the study of Baba [29], which showed that the growth rate of *S. fusiforme* propagules increased in response to elevated temperature from 10 °C to 25 °C, and was similar between 25 °C and 30 °C at an irradiance of 100 and 180 µmol photons m^−2^ s^−1^ in a 20-d experiment using nutrient-rich PESI [44] as the culture medium. The three nutrient levels were set as 10% PESI, 5% PESI, and SSW in order to evaluate the effect of reduced nutrient availability compared to natural levels. The DIN concentrations in the culture media were measured using an autoanalyzer with four replications.

In detail, matured shoots of *S. fusiforme*, in which many propagules were observed on the surface of female receptacles, were collected in June 2020 from a site in Yojiro (31°33′30″ N, 130°33′47″ E), Kagoshima Prefecture, southern Japan, and were transported to the laboratory in insulated cool boxes. These shoots were placed in a plastic container containing natural seawater for 7 d, and 54 propagules, which naturally dropped from the shoots to the container bottom, were collected using a Pasteur pipette. These propagules were placed in several petri dishes (9 mm in diameter) containing 30 mL of SSW, and were incubated at a temperature of 20 °C and an irradiance of 30 µmol photons m^−2^ s^−1^ with a 12 h light (L):12 h dark (D) photoperiod for 4 d until the start of the experiment.

The 54 propagules were assigned to one of the nine treatments. Each propagule was placed in six holes (one propagule per hole) of nine culture plates (P24F01S, AS ONE, Osaka, Japan) containing 2.5 mL of culture medium, and was incubated for 21 d at 130 µmol photons m^−2^ s^−1^ with a 12 h L:12 h D photoperiod. The culture medium was changed every 3 d. Photographs of these propagules were taken under a stereoscopic microscope using a digital camera before (initial value) and after culturing (final value), and the surface areas of all propagules were measured using Image J software [45]. The initial mean surface area (± standard deviation [SD]) was 0.096 ± 0.023 mm^−2^. Relative growth rates (% d^−1^) were calculated as 100 × ln (final value/initial value)/culture d.

All statistical analyses in the present study were performed using SPSS software version 20.0 (IBM, Armonk, NY, USA). The combined effects of temperature and nutrient availability on the relative growth rates were tested using a two-way ANOVA. When significant interactions between two factors were found, Tukey’s multiple comparison tests were used to examine the differences among the nine treatments.

### 4.2. Experiment 2: Combined Effects of Temperature and Nutrients on Holdfasts

Holdfasts of *S. fusiforme* were cultured for 28 d in eight different treatments, consisting of four temperature levels (15 °C, 20 °C, 25 °C, and 30 °C) and two nutrient levels. The four temperature levels were chosen within the temperature range at the site of collection (ca. 15 °C during winter and ca. 30 °C during summer, H. Endo unpublished data) due to the lack of available information on the optimal holdfast growth temperature. The two nutrient levels were set at 25% PESI and SSW because the holdfast growth was very slow, and was slightly affected by nutrient enrichment using 5% and 10% PESI in our preliminary experiment. The DIN concentrations in the culture media were measured in the same manner as in experiment 1.

In detail, six *S. fusiforme* individuals with relatively large holdfasts were collected in May 2018 from the site in Yojiro, and 48 holdfast segments, 5 mm in length without shoots, were cut from the specimens (eight segments per individual). Each of the eight segments derived from an individual plant were placed in a petri dish (six dishes in total) and these dishes were incubated for 24 h at 20 °C and 130 µmol photons m^−2^ s^−1^ with a 12 h L:12 h D photoperiod.

At the beginning of the experiment, the wet weight of each segment (initial value) was measured using an electronic balance (0.1 mg accuracy) after the removal of excess moisture by blotting on paper towels. The initial mean wet weight (± SD) was 19.04 ± 6.49 mg. The 48 segments were randomized into eight groups of six specimens with a similar size distribution. The eight groups were each subjected to one of the eight different treatments. These specimens were placed in a petri dish (one segment per dish) containing 30 mL of culture medium and were maintained in incubators at 130 µmol photons m^−2^ s^−1^ with a 12 h L:12 h D photoperiod for 28 d. The culture medium in each dish was changed every 7 d. At the end of the experiment, the wet weight and number of newly regenerated shoots of the cultured segment (final value) were determined. Relative growth and regeneration rates (% d^−1^) were calculated as 100 × ln (final value/initial value)/culture d. The combined effects of temperature and nutrients on the relative growth and regeneration rates were tested using two-way ANOVA and Tukey’s multiple comparison tests.

### 4.3. Experiment 3: Effect of High Temperature on Holdfasts

*Sargassum fusiforme* holdfasts were cultured at five different temperatures (30 °C, 32 °C, 34 °C, 36 °C, and 38 °C). Six *S. fusiforme* individuals were collected in July 2018 and 30 holdfast segments, 5 mm in length, were cut from the specimens (five segments per individual). These segments were incubated for 24 h at 20 °C and 130 µmol photons m^−2^ s^−1^ with a 12 h L:12 h D photoperiod. The 30 segments were randomized into five groups of six specimens with a similar size distribution. The five groups were each subjected to one of the five temperatures. These specimens were placed in a petri dish (one segment per dish) containing 30 mL of SSW and were incubated at 130 µmol photons m^−2^ s^−1^ with a 12 h L:12 h D photoperiod for 28 d. The culture medium in each dish was changed every 7 d. The wet weight and shoot number of each segment were determined before and after culturing, and the relative growth and regeneration rates were calculated in the same manner as in experiment 2. The initial mean wet weight (± SD) was 16.11 ± 3.50 mg. The effect of temperature on relative growth and regeneration rates were tested using one-way ANOVA and Tukey’s multiple comparison tests.

### 4.4. Experiment 4: Combined Effects of Fragmentation and Irradiance on Holdfasts

Holdfasts of *S. fusiforme* were cultured for 28 d in four different treatments, consisting of two fragmentation levels (cutting into five segments of 1 mm in length and a control without cutting) and two irradiance levels (30 and 130 µmol photons m^−2^ s^−1^). The two fragmentation levels were selected based on a study by Ito et al. [22], which reported that the number of regenerated shoots per unit length of holdfasts was higher for the holdfasts that were fragmented into 1-mm lengths, than for the longer holdfasts (i.e., 2.5, 5, 10, and 20 mm in length). The two irradiance levels were chosen because the relative growth rate of the holdfasts was significantly affected by reduced irradiance from 130 to 30 µmol photons m^−2^ s^−1^ in our previous experiment.

Six *S. fusiforme* individuals were collected in May 2019, and 24 holdfast segments were cut (four segments per individual). These segments were incubated for 24 h at 20 °C and 130 µmol photons m^−2^ s^−1^ with a 12 h L:12 h D photoperiod. The 24 segments were randomized into four groups of six specimens with a similar size distribution. The four groups were each subjected to one of the four treatments. These specimens were placed in a petri dish (one segment per dish) containing 30 mL of SSW and were incubated under a 12 h L:12 h D photoperiod for 28 d. The culture medium in each dish was changed every 7 d. The wet weight and shoot number of each segment were measured before and after culturing and the relative growth and regeneration rates were calculated in the same manner as in experiments 2 and 3. The initial mean wet weight (± SD) was 11.52 ± 4.43 mg. The combined effects of fragmentation and irradiance on relative growth and regeneration rates were tested using two-way ANOVA.

## Figures and Tables

**Table 1:** Results of two-way ANOVA on the effects of temperature and nutrient availability on the relative growth rate of *Sargassum fusiforme* germlings.

Source	df	MS	*F*	*p*	
Temperature (T)	2	1.385	32.408	<0.001	*
Nutrient (N)	2	2.420	56.647	<0.001	*
T × N	4	0.351	8.219	<0.001	*

* Statistical significance.

**Table 2:** Results of two-way ANOVA on the effects of temperature and nutrient availability on the relative growth and regeneration rates of *Sargassum fusiforme* holdfasts.

Source	df	MS	*F*	*p*	
Growth rate					
Temperature (T)	3	0.915	3.887	0.016	*
Nutrient (N)	1	1.129	4.797	0.034	*
T × N	3	0.052	0.222	0.881	
Regeneration rate					
Temperature (T)	3	0.254	2.478	0.075	
Nutrient (N)	1	0.100	0.973	0.330	
T × N	3	0.045	0.441	0.725	

* Statistical significance.

**Table 3:** Results of two-way ANOVA on the effects of fragmentation and irradiance on the relative growth and regeneration rates of *Sargassum fusiforme* holdfasts.

Source	df	MS	*F*	*p*	
Growth rate					
Fragmentation (F)	1	0.013	0.029	0.866	
Irradiance (I)	1	2.260	5.228	0.033	*
F × I	1	0.038	0.089	0.769	
Regeneration rate					
Fragmentation (F)	1	33.982	5.156	0.034	*
Irradiance (I)	1	0.187	0.028	0.868	
F × I	1	0.995	0.151	0.702	

* Statistical significance.
